# Understanding Verbal Violence Perpetration Among Intimate Partner Stalkers Using Police‐Identified Psychological Distress and Drug Use

**DOI:** 10.1002/cbm.70009

**Published:** 2025-09-02

**Authors:** Ebonnie Landwehr, David Garratt‐Reed, Chloe Maxwell‐Smith

**Affiliations:** ^1^ School of Population Health Curtin University Perth Australia; ^2^ Behavioural Science & Health Research Group Curtin University Perth Australia

**Keywords:** drug use, mental health, psychological distress, stalking, verbal violence

## Abstract

**Background:**

Stalkers' perpetration of verbal violence causes victims adverse mental health consequences, yet little research has examined this behaviour. Identifying correlates of verbal violence, as present in police data, could support the development of evidence‐based practical strategies for police.

**Aims:**

To understand the relationship between men's psychological distress or substance use, as identified by police, and their verbal violence against their female intimate partner stalking victims.

**Methods:**

Data were extracted from anonymised police records of 603 men in Western Australia linked to a stalking offence in relation to a woman who is or was their intimate partner. Logistic regression analyses were used to examine whether police‐identified psychological distress or drug use, as described in police incident reports, was significantly associated with verbal violence perpetration. Separate models were used to assess different expressions of verbal violence: ‘death/injury/sexual assault threats’ to the victim or of ‘other verbal violence’ only, and of the binary aggregate of these groups.

**Results:**

Police‐identified psychological distress (OR = 2.3–3.1) and drug use (OR = 2.1–2.2) were independently associated with reported verbal violence perpetration. Different patterns of findings emerged when verbal violence expression was differentiated across four contrasting groups. The model comparing ‘death/injury/sexual assault threats’ with ‘no verbal violence recorded’ explained over four times the variance of the model comparing ‘other verbal violence only’ with ‘no verbal violence recorded’. Physical violence was also relevant for understanding verbal violence perpetration.

**Conclusions:**

This study is the first to explore correlates of intimate partner stalkers' verbal violence perpetration. According to police records, verbal violence was differentially associated with psychological distress, drug use and physical violence according to type of verbal violence and comparison group. These differences suggest that verbal violence expression types should be considered separately when intervening and within research designs.

## 1 Introduction

Stalking is a legal term that refers to intrusive interpersonal behaviour. Although legislation varies between jurisdictions, the elements of a stalking offence generally reflect (1) the perpetrator having engaged in repeated unwanted actions and (2) the victim feeling intimidated by the perpetrator's actions or the perpetrator having *intended* to intimidate their victim (Davis et al. 2012). Notably, although this definition does not require the victim to be physically harmed by the perpetrator, it highlights the victim's experience in reaction to the intrusive behaviour. Thus, stalking is a form of psychological violence, and victims frequently suffer emotional distress as a result, independently of experiencing any physical violence (Logan 2022), but there has been little scholarly investigation of psychological violence against women (Alkan et al. 2022; Dokkedahl et al. 2022).

There is no consensus on defining psychological violence, which can encompass stalking, coercive control, property damage and verbal violence (Free and Beck 2024; Follingstad 2007). Verbal violence is an umbrella term for acts including death threats, verbal abuse, insulting and/or belittling the victim (Free and Beck 2024). Research shows that 30%–73% of stalking situations involve threats (Bendlin and Sheridan 2021; Brewster 2000; Mullen et al. 1999) and 54%–95% involve criticism or insults (Baldry et al. 2016; Roberts 2005). Ex‐intimate partner stalking victims experience more threats than victims of stranger and acquaintance stalking (Logan 2020; Palarea et al. 1999). Despite the extent of verbal violence perpetrated by stalkers, there is little research examining this behaviour in a stalking context, other than the more specific concept of threats as predictors of physical violence (Churcher and Nesca 2013; Groenen and Vervaeke 2009; T. McEwan et al. 2007; T. E. McEwan et al. 2017; Thompson et al. 2020).

There is value in understanding verbal violence perpetration among stalkers, especially those who come to the attention of legal authorities and are likely to have caused significant harm to their victim(s) (Cho et al. 2023; Reyns and Englebrecht 2010). Although not all stalkers who use verbal violence go on to use physical violence, the majority of those who are physically violent have also made threats (Churcher and Nesca 2013; T. McEwan et al. 2007). Furthermore, both intimate partner stalking and death threats are risk factors for intimate partner homicide (Matias et al. 2020). Threats are associated with victim fear (Logan 2022), and victim fearfulness is positively related to the prosecutorial decision to proceed with a stalking case (Brady and Reyns 2020). It is therefore crucial to understand the expression of verbal violence to inform the legal process and as an early intervention point before a situation escalates with potentially fatal consequences.

In nonstalking samples, verbal violence has been linked to the perpetrator's psychological distress and substance use (Dutton and Karakanta 2013; Glassman et al. 2021; Ruchkin et al. 2023; Shorey et al. 2012; Tomlinson et al. 2016). Psychological distress is a broad term capturing stress, symptoms of mental disorder and psychiatric diagnoses (Zhu et al. 2022). Many stalking victims rely on court‐issued protective orders to restrain their stalker's behaviour. Although these orders reduce serious physical violence perpetration, they are much less effective in managing the behaviour of individuals who engage in nonphysical violence or have a history of psychological distress (Dowling et al. 2018).

Although the extent of substance use among stalkers is not clear (Landwehr et al. 2024), it is a common behaviour among those who perpetrate intimate partner violence and threats to kill (Warren et al. 2008; Warren et al. 2011; Willson et al. 2000) and is linked with physical violence perpetration among intimate partner stalkers (Landwehr et al. 2025; Rosenfeld 2004). Understanding the correlates of verbal violence perpetration by stalkers can improve identification of those who engage in the behaviour.

Our aim was to explore the extent to which men's use of verbal violence against their female intimate partner stalking victims is associated with these men's experiences of psychological distress and/or substance use, as described within police stalking incident reports.

## 2 Method

### 2.1 Ethical Approval and Data Availability

The Curtin University Human Research Ethics Committee provided approval for this study (HRE2019‐0709). Because of the sensitive nature of the data used in this study, they are not publicly available.

### 2.2 Dataset

This study was conducted within a larger project on intimate partner stalking, and the method has been described in detail by Landwehr et al. (2025). The Western Australia Police Force provided anonymised data relating to all incident reports occurring between 01/01/2005 and 31/12/2014 where the offence of stalking was recorded (*N* = 1826). One duplicate record was identified and excluded (*N* = 1825). The Western Australia Criminal Code proscribes stalking as a crime, defined in s338E(1) as ‘a person who pursues another person with intent to intimidate that person or a third person’, and as a simple offence, defined in s338E(2) as ‘a person who pursues another person in a manner that could reasonably be expected to intimidate, and that does in fact intimidate, that person or a third party’. Incident reports involving a male perpetrator and female victim who currently or had at one time shared an intimate relationship (*N* = 704) met inclusion for this study.

Incident reports were reviewed to ascertain the perpetrator‐victim sex and perpetrator–victim relationship. We accepted the sex designated in the police record—invariably male or female—for each of the 1826 incident reports, as gender identity was not recorded. Where the relationship type could not be unequivocally determined, the incident report was excluded (*N* = 517). Relationship types other than intimate partners (e.g., acquaintance, stranger, friend, work contacts) were excluded (*N* = 534). Of the remaining 774 incident reports for intimate partners (e.g., married, de facto, sexual relationship, dating), only those describing a man pursuing a woman were retained for this study (*N* = 704). Data were examined at the perpetrator level (*N* = 603). Most men had a single victim (*N* = 588, 98%) and were recorded as an alleged perpetrator in either one (*N* = 527, 87%) or two (*N* = 61, 10%) stalking incident reports. Study variables were created on the basis that the perpetrator was described as engaging in a behaviour with *at least one* stalking victim in *any* stalking incident report.

As the incident reports were selected on the presence of a *stalking offence*, both suspects and individuals whose guilt was later determined in court were included. Stalking case attrition is an established problem; police infrequently charge, prosecution is difficult, and convictions are rare (Backes et al. 2020; Brady and Nobles 2017; Brady and Reyns 2020; Jordan et al. 2003; Lynch and Logan 2015; Ngo 2019; Storey and Hart 2011); therefore, samples of charged or convicted men mostly represent extreme cases. Examining incident reports for an alleged *stalking offence* centralises the victim's experience rather than police outcomes (e.g., insufficient evidence to prosecute).

#### 2.2.1 Incident Report Information

Within the overarching incident report, police record their notes and observations of a situation and the involved parties in an open‐text narrative (Karystianis et al. 2024). In addition, in Western Australia, police officers are required to complete a family violence incident report form (FVIR; active from 18/08/2013) for incidents involving parties who are or were in a family relationship where actual or threatened violence is alleged. The FVIR form contains an additional open‐text narrative, checkboxes for known risk factors (e.g., threats, mental health issues), which police can record their observations against, and qualitative fields for elaboration (Bendlin and Sheridan 2021). Overall, few stalking incidents in this study included an FVIR (*N* = 77, 11%), but of the 85 stalking incident reports recorded after the FVIR implementation date, most included an FVIR (*N* = 77, 90%). Data examined in this study were from these stalking incident report narratives and FVIR form, if completed, including sex of the perpetrators and victims; police identification numbers were not used in the main database.

### 2.3 Variables

All variables were coded from information recorded in the police incident reports. This came primarily from the qualitative open‐text narratives; however, information was also collated from checkbox and qualitative field data where a family violence incident report was included. The dependent variable in this study, *verbal violence*, was rated as present if there was evidence of threats, abuse and/or insults communicated directly to the victim (including written material and texts) and that was about the victim or a third party important to the victim (e.g., children). If there was no evidence of any communication of this type directed towards the woman, the behaviour was rated as absent. Evidence could be from the perpetrator or victim report (e.g., victim: ‘he said he would make me regret this’) or observation by the recording police officer (e.g., the perpetrator made threats against the victim). Categories of *verbal violence* were generated by splitting this group into two mutually exclusive subgroups by type of verbal violence, simply coded present/absent:

1. ‘Serious threat to kill/injure/sexually assault victim’ or
2. ‘Other verbal violence’.

Isolating threats of death, injury or sexual assault to the victim was the primary interest, as serious threats are associated with intimate partner homicide (Bridger et al. 2017; Matias et al. 2020), perpetrator suicide (Warren et al. 2008) and life‐threatening physical violence such as nonfatal strangulation (Stansfield and Williams 2021; Thomas et al. 2014). A minority of cases in the present sample described threats of harm to a victim’s child, and all co‐occurred with a threat of physical harm to the victim. The ‘threat to kill/injure/sexually assault victim’ category was constructed first, then all remaining verbal violence was collapsed to a single group. As such, ‘other verbal violence’ refers to a heterogeneous category encompassing verbal violence that did not include a death, injury or sexual assault threat to the victim or their child. Examples include the following: verbal abuse (e.g., insults, shouting); verbal violence towards third parties where there was no death, injury or sexual assault threat to the victim/victim’s child; threats about damaging the victim’s property or reputation; threats to be a persistent presence in the victim’s life; and nonspecified threats described in the narrative (e.g., ‘threatened the victim’, ‘sent intimidating texts’).

The first author coded all incident reports to obtain the variables used in this study. Two researchers rated a random sample of incident reports (*N* = 80), blind to each other, to determine if verbal violence was present and to classify it as above. There was good agreement between them (Cohen’s kappa *κ* = 0.9; see MacPhail et al. 2016). The few disagreements were resolved by discussion and consensus.

The independent variables were perpetrator *psychological distress, drug use* and *alcohol use.* Using an approach similar to that of Karystianis et al. (2018), *psychological distress* (1 = identified, 0 = not identified) was a composite, binary variable including any evidence of any of the following: negative mood state (e.g., depression), suicidality, use of any prescribed psychotropic medication (e.g., antidepressants) and unspecified mental health problems. *Drug use* (1 = identified, 0 = not identified) and *alcohol use* (1 = identified, 0 = not identified) included descriptions of addiction, intoxication, misuse and use of specific substances (e.g., alcohol, methamphetamine, cannabis) described in the incident report. The variable *severity of physical violence,* included as a covariate, captured physical violence described in the incident report (severe = 2, moderate = 1, not identified = 0). For consistency with existing research, this variable was categorised according to the work of Bendlin and Sheridan (2021), who adapted the conflict tactics scale (Straus et al. 1996). Severe physical violence included punching, choking and weapon use, whereas moderate violence included pushing and spitting. For further detail see Landwehr et al. (2025) and Appendix A. Because of the nature of the data being examined, the independent variables represent only the presence of a factor, not the proximity between a factor and the stalking episode, as this was generally not possible to determine.

### 2.4 Analytical Plan

Bivariate relationships between the study variables were assessed using chi‐square statistics. Independent variables that were significantly related at this binary level were included in the regression models.

Four binary logistic regression models were created, allowing for differences in type of verbal violence. Model 1 included all the men, taking any evidence of verbal violence (vs. none) as the dependent variable. The other models examined subgroups as follows: Model 2 compared men who made death, injury or sexual assault threats *towards the victim* with verbally nonviolent men; Model 3 compared men who were verbally violent but did not make death, injury or sexual assault threats towards the victim with verbally nonviolent men; and Model 4 compared the two verbal violence groups (excluding the verbally nonviolent men).

Model 1 included the potentially explanatory variables *psychological distress, drug use* and *severity of perpetrated physical violence* as the independent variables; alcohol use was excluded as it was not significant at the binary level. *Alcohol use* was only significantly associated with death/injury/sexual assault threats and so was excluded from the main regression models reported in the results; regression models for death/injury/sexual assault threats that include *alcohol use* are provided in Appendix B (Tables A2 and A3). Including *alcohol use history* decreased the robustness of analyses, as 29%–50% of cells had an expected cell count below 5 across the four models. This exceeds the established guideline of no more than 20% (Tabachnick and Fidell 2014). We opted to ensure the assumptions of logistic regression were met across models and excluded alcohol for consistency and simplicity when interpreting the results. Relationships between independent and dependent variables were expressed in terms of odds ratios and confidence intervals. Significance was set to an alpha value of 0.05. All analyses were run using Statistical Package for the Social Sciences (SPSS) software (version 28.0).

## 3 Results

### 3.1 General Description of the Sample

Table 1 shows the distributions of the relevant variables. Verbal violence had been recorded in just over half the sample (*N* = 327, 54%) and physical violence for one third (*N* = 196, 33%). Psychological distress, drug use and alcohol use were reported for a minority of men in the sample.

**TABLE 1:** Overall frequencies (*N* = 603).

Incident report (IR) data	Level	*N*	%
Perpetrator psychological distress in IR	Recorded	85	14.1
	Not recorded	518	85.9
Perpetrator drug use in IR	Recorded	71	11.8
	Not recorded	532	88.2
Perpetrator alcohol use in IR	Recorded	49	8.1
	Not recorded	554	91.9
Physical violence in IR	Recorded	196	32.5
	Not recorded	407	67.5
Severity of physical violence in IR	Severe	84	13.9
	Moderate	112	18.6
	Not recorded	407	67.5
Verbal violence in IR	Recorded	326	54.1
	Not recorded	277	45.9
Verbal violence in IR group	Death/injury/sexual assault threat	161	26.7
	Other verbal violence	165	27.4
	Verbal violence not recorded	277	45.9
Physical violence in IR and severe threat in IR	Recorded	79	13.1
	Not recorded	524	86.9
Physical violence in IR by verbal violence in IR	Physical and verbal violence	123	20.4
	Verbal violence only	203	33.7
	Physical violence only	73	12.1
	Neither recorded	204	33.8

### 3.2 Bivariate Relationships Between Verbal Violence and Characteristics of the Men

Table 2 shows relationships between verbal violence and each independent variable in turn. Although psychological distress was consistently associated with verbal violence, the other variable relationships differed depending on verbal violence type.

**TABLE 2:** Univariate statistics across four models of verbal violence.

Model 1		Verbal violence recorded (*N* = 326)		Verbal violence not recorded (*N* = 277)		*χ* ^2^	*p*
		*n*	%	*n*	%		
PD	Recorded	63	19.3	22	7.9	16.02	< 0.001
	Not recorded	263	80.7	255	92.1		
Drugs	Recorded	52	16.0	19	6.9	11.92	< 0.001
	Not recorded	274	84.0	258	93.1		
Alcohol	Recorded	32	9.8	17	6.1	2.72	0.099
	Not recorded	294	90.2	260	93.9		
Severity physical	Severe	55	16.9	29	10.5	9.27	0.010
	Moderate	68	20.9	44	15.9		
	Not recorded	203	62.3	204	73.6		

Model 2		Death/injury/sexual assault threat (*N* = 161)		Verbal violence not recorded (*N* = 277)		*χ* ^2^	*p*
		*n*	%	*n*	%		
PD	Recorded	39	24.2	22	7.9	22.52	< 0.001
	Not recorded	122	75.8	255	92.1		
Drugs	Recorded	27	16.8	19	6.9	10.64	0.001
	Not recorded	134	83.2	258	93.1		
Alcohol	Recorded	19	11.8	17	6.1	4.33	0.037
	Not recorded	142	88.2	260	93.9		
Severity physical	Severe	38	23.6	29	10.5	24.34	< 0.001
	Moderate	41	25.5	44	15.9		
	Not recorded	82	50.9	204	73.6		

Model 3		Other verbal violence only recorded (*N* = 165)		Verbal violence not recorded (*N* = 277)		*χ* ^2^	*p*
		*n*	%	*n*	%		
PD	Recorded	24	14.5	22	7.9	4.84	0.028
	Not recorded	141	85.5	255	92.1		
Drugs	Recorded	25	15.2	19	6.9	7.93	0.005
	Not recorded	140	84.8	258	93.1		
Alcohol	Recorded	13	7.9	17	6.1	0.50	0.481
	Not recorded	152	92.1	260	93.9		
Severity physical	Severe	17	10.3	29	10.5	0.02	0.991
	Moderate	27	16.4	44	15.9		
	Not recorded	121	73.3	204	73.6		

Model 4		Death/injury/sexual assault threat (*N* = 161)		Other verbal violence only recorded (*N* = 165)		*χ* ^2^	*p*
		*n*	%	*n*	%		
PD	Recorded	39	24.2	24	14.5	4.90	0.027
	Not recorded	122	75.8	141	85.5		
Drugs	Recorded	27	16.8	25	15.2	0.60	0.690
	Not recorded	134	83.2	140	84.8		
Alcohol	Recorded	19	11.8	13	7.9	1.42	0.234
	Not recorded	142	88.2	152	92.1		
Severity physical	Severe	38	23.6	17	10.3	18.35	< 0.001
	Moderate	41	25.5	27	16.4		
	Not recorded	82	50.9	121	73.3		

Abbreviations: Alcohol, perpetrator's alcohol use mentioned in the incident report; drugs, perpetrator's drug use mentioned in the incident report; PD, perpetrator's psychological distress mentioned in the incident report; severity physical, severity of physical violence by the perpetrator recorded in the incident report.

### 3.3 Binary Logistic Regression Modelling

All four models had significant chi‐square goodness‐of‐fit tests (see Table 3), but each model differed in the amount of variance explained, as well as the combination of variables, that explained verbal violence.

**TABLE 3:** Binomial regression models for verbal violence variables.

	*β*	SE	Wald	df	*p*	OR	95% CI		Model statistics	
							Lower	Upper	Significance	*R* ^2^
Model 1									*χ* ^2^ (4) = 30.66, *p* < 0.001	6.6%
Psychological distress in IR	0.846	0.269	9.878	1	0.002	2.33	1.38	3.95		
Drug use in IR	0.754	0.290	6.772	1	0.009	2.13	1.20	3.75		
Severity of physical violence in IR			5.610	2	0.061					
Moderate	0.323	0.223	2.091	1	0.148	1.38	0.89	2.14		
Severe	0.539	0.255	4.453	1	0.035	1.71	1.04	2.83		
Model 2									*χ* ^2^ (4) = 47.07, *p* < 0.001	13.9%
Psychological distress in IR	1.135	0.300	14.351	1	< 0.001	3.11	1.73	5.60		
Drug use in IR	0.759	0.335	5.114	1	0.024	2.14	1.12	4.12		
Severity of physical violence in IR			18.765	2	< 0.001					
Moderate	0.751	0.262	8.211	1	0.004	2.12	1.27	3.55		
Severe	1.102	0.287	14.758	1	< 0.001	3.01	1.72	5.28		
Model 3									*χ* ^2^ (4) = 10.87, *p* = 0.028	3.3%
Psychological distress in IR	0.574	0.321	3.192	1	0.074	1.78	0.95	3.33		
Drug use in IR	0.812	0.328	6.129	1	0.013	2.25	1.18	4.28		
Severity of physical violence in IR			0.124	2	0.940					
Moderate	−0.065	0.277	0.055	1	0.815	0.94	0.55	1.61		
Severe	−0.099	0.334	0.088	1	0.767	0.91	0.47	1.74		
Model 4									*χ* ^2^ (4) = 21.16, *p* < 0.001	8.4%
Psychological distress in IR	0.483	0.306	2.499	1	0.114	1.62	0.89	2.95		
Drug use in IR	−0.148	0.325	0.207	1	0.649	0.86	0.46	1.63		
Severity of physical violence in IR			15.553	2	< 0.001					
Moderate	0.762	0.291	6.861	1	0.009	2.14	1.21	3.79		
Severe	1.143	0.329	12.090	1	< 0.001	3.14	1.65	5.97		

*Note:* The comparator groups among the explanatory variables were as follows: psychological distress not recorded in the IR, drug use not recorded in the IR and physical violence not recorded in the IR. Model 1 compared ‘verbal violence recorded’ with ‘verbal violence not recorded’. Model 2 compared ‘death/injury/sexual assault threat’ with ‘verbal violence not recorded’. Model 3 compared ‘other verbal violence only recorded’ with ‘verbal violence not recorded’. Model 4 compared ‘death/injury/sexual assault threat’ with ‘other verbal violence only recorded’.

Abbreviations: CI, confidence interval; IR, incident report; OR, odds ratio; *R* ^2^, Nagelkerke statistic.

Men who were recorded as having been verbally violent to their victim were over twice as likely as men who had not been verbally violent to have been described as psychologically distressed (Model 1; OR = 2.33, CI 1.38–3.95) or as drug users (OR = 2.13, CI 1.20–3.75). Physical violence was nonsignificant in the model.

Men who were recorded as having made death, injury or sexual assault threats to their victim were three times more likely than men who had not been verbally violent (Model 2) to have been described as psychologically distressed (OR = 3.11, CI 1.73–5.60) or severely physically violent to their victim (OR = 3.01, CI 1.72–5.28) and twice as likely to be described as a drug user (OR = 2.14, CI 1.12–4.12) or moderately physically violent to their victim (OR = 2.12, CI 1.27–3.55).

Men who were recorded as having been verbally violent towards their victim in a manner other than threatening death, injury or sexual assault to the victim were twice as likely as men who had not been verbally violent (Model 3) to have been described as a drug user (OR = 2.25, CI 1.18, 4.28). Psychological distress and physical violence were nonsignificant in the model.

Men who were recorded as having made death, injury or sexual assault threats to their victim were more likely than men who used other types of verbal violence (Model 4) to be described as severely (OR = 3.14, CI 1.65, 5.97) or moderately (OR = 2.14, CI 1.21, 3.79) physically violent. Neither psychological distress nor drug use was significant in the model.

## 4 Discussion

We examined police data to establish the extent to which men's experiences of psychological distress or substance use, as reported in stalking incident reports, were related to verbal violence perpetration against the female intimate partner whom they were (allegedly) stalking. Overall, both psychological distress and drug use increased the odds of verbal violence perpetration, at least as recorded in the police narratives. When the verbal violence was differentiated to examine men who made threats to kill, injure or sexually assault their victim separately from those who used other types of verbal violence, two patterns emerged. Drug use more than doubled the odds of the perpetrator being in either group compared with those who had no verbal violence recorded—but did not differentiate between the men in the two verbal violence groups. Psychological distress was associated with about three times the odds of a perpetrator having made threats to kill, injure or sexually assault the victim recorded rather than no verbal violence, but did not distinguish between the two verbal violence groups. Only actual physical violence differentiated the two verbal violence groups, being more likely in the threats of death, injury or sexual assault group.

Our findings suggest potential value in distinguishing different expressions of verbal violence, which is consistent with other research accounting for the severity of stalkers' physical violence perpetration (James and Farnham 2003; Landwehr et al. 2025; Sheridan and Roberts 2011; Thompson et al. 2013). Variations in definitions of verbal violence are, however, likely to influence discrepancies in the literature. For example, compared with previous analyses of threats within police stalking data, we found a higher prevalence of verbal violence than Bendlin and Sheridan (25%; 2019) but a substantially lower proportion than Groenen and Vervaeke (67%; 2009). In our study, the regression model for threats of death, injury or sexual assault to the victim versus no verbal violence recorded explained four times more variance than its counterpart for other types of verbal violence. We therefore caution against aggregation of all verbal violence into a single factor when exploring risk.

### 4.1 Policing Implications

Given that verbal violence may cause significant psychological injury to victims (Dokkedahl et al. 2022), police responding to stalking incidents should not only assess injuries and property damage, but also nonphysical forms of violence. Victims may not voluntarily disclose verbal violence if they deem it to fall short of the threshold for harm required for police intervention (Taylor‐Dunn et al. 2021). Our preliminary findings, however, suggest that a police‐identified history of substance use or psychological distress may be a prompt to ask the victim about their experience of verbal violence by the perpetrator and, in particular, the nature of any explicit threats. We acknowledge that police officers across Australian jurisdictions and internationally face complexity when responding to intimate partner violence (Backes et al. 2020; Saxton et al. 2022) while also urging that more needs to be done to improve victim outcomes, but police officers' exploration of the alleged perpetrator's drug use and psychological distress during their investigation can aid with assessing victim fear, which is relevant for prosecutorial decisions (Brady and Reyns 2020), and the appropriateness of referring the perpetrator to a clinical service.

### 4.2 Strengths, Limitations and Future Research

Our results support practically relevant implications for law enforcement, as although police data may not reflect clinical perspectives, this is the information known to and deemed relevant by police officers at the time they respond to an incident. In this study, a large minority of the incident reports did not record verbal violence, and it is possible that instances of verbal violence were not captured by responding police officers, though investigative case files (not examined in this study) would contain greater detail. The proportion of incident reports describing verbal violence may also reflect the fact that a stalking offence refers to an *episode* of behaviour that can involve many and varied behaviours—men in the study sample who were not verbally violent may have engaged in other tactics that were not examined.

It is not clear *how* officers decide the relevance of information they record, a limitation requiring further study. Nonetheless, police data may improve the evidence base in this area, given that past research has shown offenders minimise their substance use and self‐harm when self‐reporting (Borschmann et al. 2017; Flowers et al. 2020). Alcohol use history is almost certainly under‐represented, as it was identified across our sample at a level lower than indicated by population surveys in the community the data represent (Australian Institute of Health and Welfare 2024a, 2024b; AIHW). Culturally, heavy drinking is common in Australia, and, compared with illicit drugs, problematic consumption of alcohol may be less identified by the men themselves (Kissell et al. 2014) and/or police officers. Although half of Australian men have used illicit drugs at some point in their lives (AIHW 2024a, 2024b), the extent to which their use has resulted in legal consequences is unclear.

This study is correlational and cannot establish causal pathways between stalker characteristics and behaviours, yet the findings contribute to expanding theoretical understanding of stalking behaviour and support further examination of psychological risk factors that may underlie stalkers' problematic behaviours. It is likely that the mechanisms underpinning different expressions of verbal violence (e.g., insults vs. death threats) vary depending on other individual and environmental factors. Therefore, future research on verbal violence perpetration may explore the roles of motivation (e.g., revenge/punishment vs. maladaptive reconciliation attempts), empathy, alexithymia and biophysiological changes to behaviour associated with long‐term illicit drug use.

We were interested in examining men who made threats to kill, injure or sexually assault their victim separately from men who used other types of verbal violence. As the ‘other’ group was heterogeneous, we encourage future research that further distinguishes types of violence, such as separating insults from threats to damage reputation.

## 5 Conclusions

Research has typically focused on physical violence outcomes of stalking. To our knowledge, this is the first study to explore intimate partner stalkers' use of verbal violence. Verbal violence perpetration was associated with greater odds of police‐identified perpetrator psychological distress and drug use recorded during inquiry into stalking incidents. These factors, together with physical violence in the incident report, provided the strongest potential explanation of verbal violence. Our results suggest that further exploration of verbal violence and its correlates could have value in preventing escalation towards physical violence if victims and police alike are aware of its correlates.

## Ethics Statement

The Curtin University Human Research Ethics Committee provided approval for this study (HRE2019‐0709).

## Conflicts of Interest

The authors declare no conflicts of interest.

## Data Availability

Because of the sensitive nature of the data examined in this study, they are not publicly available.

## Appendix A

Table A1

**TABLE A1:** Classification of behaviours—physical violence severity.

Severity	Behaviour
Moderate	Threw something at my partner that could hurt
	Pulled my partner's hair
	Pushed or shoved my partner
	Grabbed my partner
	Slapped my partner
	Restrained or prevented the victim from leaving
	Possessed a weapon
	Violence/assault not otherwise specified
	Spat on victim
Severe	Used a weapon on my partner
	Punched or hit my partner with something that could hurt
	Choked my partner
	Threw my partner against something
	Beat up my partner or broke a bone
	Burnt or scalded my partner on purpose
	Kicked my partner
	Used a vehicle aggressively
	Prevented my partner from breathing
	Stuck fingers in my partner's eyes
	Bit partner
	Pinned my partner down by the throat
	Refused my partner medical attention
	Solicited to murder my partner
	Murdered my partner

## Appendix B

Table A2

**TABLE A2:** Binomial regression model—alternate Model 2 including alcohol use.

	*β*	SE	Wald	df	*p*	OR	95% CI	
							Lower	Upper
Psychological distress in IR	1.133	0.304	13.927	1	< 0.001	3.11	1.71	5.63
Drug use in IR	0.754	0.354	4.533	1	0.033	2.13	1.06	4.25
Alcohol use in IR	0.018	0.406	0.002	1	0.965	1.02	0.46	2.26
Physical violence in IR			18.695	2	< 0.001			
Moderate	0.750	0.264	8.108	1	0.004	2.12	1.26	3.55
Severe	1.102	0.287	14.754	1	< 0.001	3.01	1.72	5.28

*Note:* The regression model was significant, *χ* ^2^ (5) = 47.08, *p* < 0.001. Nagelkerke *R* ^2^ = 13.9%. The model compares ‘severe threat recorded’ with ‘no verbal violence recorded’.

Table A3

**TABLE A3:** Binomial regression model—alternate Model 4 including alcohol use.

	*β*	SE	Wald	df	*p*	OR	95% CI	
							Lower	Upper
Psychological distress in IR	0.457	0.309	2.193	1	0.139	1.58	0.86	2.89
Drug use in IR	−0.197	0.336	0.346	1	0.556	0.28	0.43	1.59
Alcohol use in IR	0.252	0.411	0.374	1	0.541	1.29	0.57	2.88
Physical violence in IR			15.286	2	< 0.001			
Moderate	0.757	0.291	6.771	1	0.009	2.13	1.21	3.77
Severe	1.134	0.329	11.870	1	< 0.001	3.11	1.63	5.92

*Note:* The regression model was significant, *χ* ^2^ (5) = 21.54, *p* < 0.001. Nagelkerke *R* ^2^ = 8.5%. The model compares ‘severe threat recorded’ with ‘other verbal violence only recorded’.
